# Catching a glimpse of the bacterial gut community of companion animals: a canine and feline perspective

**DOI:** 10.1111/1751-7915.13656

**Published:** 2020-08-30

**Authors:** Giulia Alessandri, Chiara Argentini, Christian Milani, Francesca Turroni, Maria Cristina Ossiprandi, Douwe van Sinderen, Marco Ventura

**Affiliations:** ^1^ Department of Veterinary Medical Science University of Parma Parma Italy; ^2^ Laboratory of Probiogenomics, Department of Chemistry, Life Sciences, and Environmental Sustainability University of Parma Parma Italy; ^3^ Microbiome Research Hub University of Parma Parma Italy; ^4^ APC Microbiome Institute and School of Microbiology, Bioscience Institute National University of Ireland Cork Ireland

## Abstract

Dogs and cats have gained a special position in human society by becoming our principal companion animals. In this context, efforts to ensure their health and welfare have increased exponentially, with in recent times a growing interest in assessing the impact of the gut microbiota on canine and feline health. Recent technological advances have generated new tools to not only examine the intestinal microbial composition of dogs and cats, but also to scrutinize the genetic repertoire and associated metabolic functions of this microbial community. The application of high‐throughput sequencing techniques to canine and feline faecal samples revealed similarities in their bacterial composition, with Fusobacteria, Firmicutes and Bacteroidetes as the most prevalent and abundant phyla, followed by Proteobacteria and Actinobacteria. Although key bacterial members were consistently present in their gut microbiota, the taxonomic composition and the metabolic repertoire of the intestinal microbial population may be influenced by several factors, including diet, age and anthropogenic aspects, as well as intestinal dysbiosis. The current review aims to provide a comprehensive overview of the multitude of factors which play a role in the modulation of the canine and feline gut microbiota and that of their human owners with whom they share the same environment.

## Graphical abstract

Dogs and cats have gained a special position in human society by becoming our principal companion animals. In this context, efforts to ensure their health and well‐fare have increased exponentially, with in recent times a growing interest in assessing the impact of the gut microbiota on canine and feline health. Recent technological advances have generated new tools to not only examine the intestinal microbial composition of dogs and cats, but also to scrutinize the genetic repertoire and associated metabolic functions of this microbial community, revealing that the taxonomic composition and the metabolic repertoire of the intestinal microbial population of dogs and cats may be influenced by several factors, including diet, age and anthropogenic aspects, as well as intestinal dysbiosis.

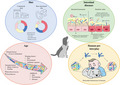

## Introduction

Being descendants of the Eurasian grey wolf and wild cat, domesticated dogs and cats, respectively, are among the first animals to have undergone profound anthropogenic changes (Sykes *et al*., 2020). Since their initial domestication, a process that commenced some 15 000 years ago, a large number of canine and feline breeds have been selected with an associated global dissemination of millions of these pets. Throughout recent millennia, dogs and cats have played an increasingly important role in human society. The ever‐closer relationship between these pets and their owners resulted in the former becoming the principal companion animals for humans. Furthermore, intense urbanization typical of the modern era has not only changed human habits, but has also completely altered the lifestyle of these pets (Dotson and Hyatt, 2008). In this context, concerns regarding pet health and well‐being are taken seriously and have consequently prompted a lot of research aimed at pet health promotion and disease prevention. In this context, the scientific community has in recent decades focused on gut health and the study of the intestinal bacterial population of dogs and cats, for the purpose of maintaining and promoting host health (Mondo *et al*., 2019).

The mammalian gastrointestinal tract (GIT) is inhabited by one of the most intricate and diverse communities of microorganisms in the biosphere, i.e. the gut microbiota, encompassing bacteria, archaea, viruses, fungi and protozoa (Suchodolski, 2011). Despite this diversity of microorganisms that may colonize the mammalian intestine, bacteria are by far the most abundant representatives of the mammalian intestinal population (Suchodolski, 2011, 2016). In this context, the long‐lasting mutualistic association (commensalism or symbiosis) of this indigenous microbial ecosystem with its mammalian host has laid the foundation for the establishment and subsequent consolidation of multiple trophic interactions. Specifically, the intestinal microbial community is involved in various metabolic and physiological activities, including degradation of otherwise non‐digestible complex carbohydrates, provision of energy sources to support intestinal epithelium integrity and to ensure its basal activity, education of the immune system, protection against pathogen colonization and production of metabolites, including short‐chain fatty acids (SCFAs), secondary bile acids, vitamins or other bacterially derived compounds such as trimethylamine‐N‐oxide (Moens and Veldhoen, 2012; Pickard *et al*., 2017; Moon *et al*., 2018; Mondo *et al*., 2019; Wernimont *et al*., 2020). Furthermore and similar to the human situation, the microbial community of the feline and canine intestine may be influenced by various factors, such as diet (Schmidt *et al*., 2018), age (Masuoka *et al*., 2017), metabolic disorders and inflammatory bowel diseases (Omori *et al*., 2017; Kalenyak *et al*., 2018). Despite the multiple functions exerted by the intestinal ecosystem and its involvement in supporting host health, our understanding of the gut microbiota of pet animals is rather scarce if at best incomplete when compared to the significant scientific progress achieved for similar studies of the human gut microbiota. In the present review, current knowledge on canine and feline intestinal community will be analysed. More specifically, we will discuss the composition of the gut microbiota of healthy dogs and cats, together with perturbations that this microbial community may undergo as a consequence of the onset of inflammatory bowel diseases, age or changes in dietary habits. Furthermore, we will discuss the impact of the close human–pet relationship on the microbiota of either party.

## Overview of technical approaches for gut microbiota characterization

Originally, the study of canine and feline gut microbiota was based on culture‐dependent methods, involving cultivation and subsequent isolation of microorganisms by means of different growth media (Moon *et al*., 2018). Bacterial cultivation was typically used to detect specific enteropathogens, i.e. *Salmonella* spp. or *Campylobacter* spp., to determine active infection or to test for antibiotic sensitivity (Johnston *et al*., 2001; Pepin‐Puget *et al*., 2020). However, despite the advantages of these classical microbiological techniques, such as the possibility to perform physiological and biochemical analyses on isolated strains or to assess total viable bacterial load of a sample, culture‐dependent methods suffer from several disadvantages (Turroni *et al*., 2008). Indeed, samples require immediate processing and results are very much affected by cultivation media and laboratory conditions used, which are unlikely to faithfully reproduce the very complex intestinal environment. In this context, it has been stated that only a small portion of the intestinal biodiversity can be assessed by the application of culture‐dependent methods (Furrie, 2006; Suchodolski, 2016). However, in recent times these limitations have been overcome thanks to remarkable advances in sequencing technologies, opening up new research horizons to investigate ecological aspects of the gut microbiota and to perform culture‐independent approaches by means of cost‐effective, high‐throughput sequencing methods, such as metagenomics and metatranscriptomics. Furthermore, technological developments in metaproteomics and metabolomics areas have allowed researches to connect DNA sequences of the gut microbiota with their encoded functions.

**Fig. 1:**
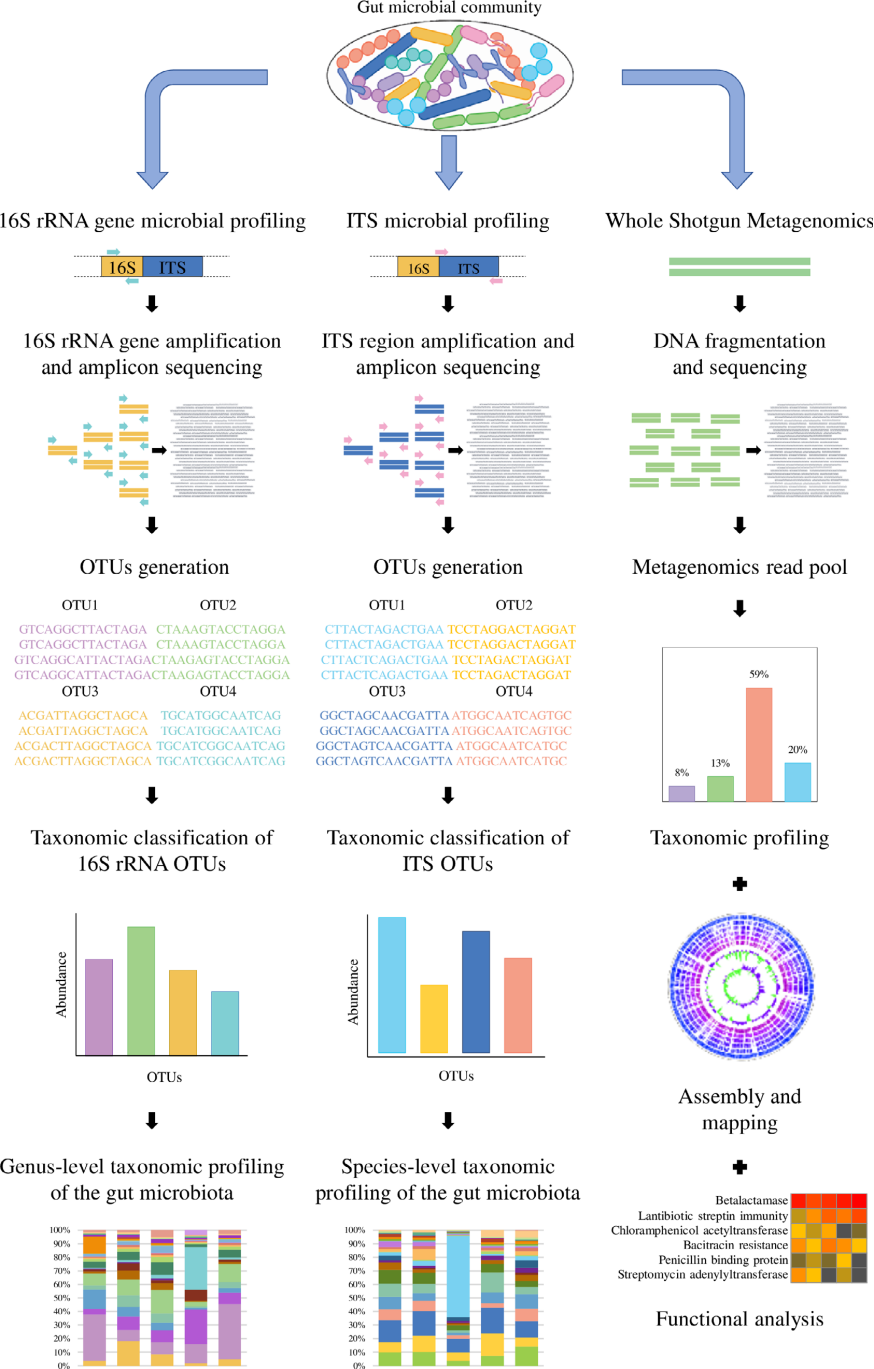
General overview of the metagenomic approaches available for gut microbiota characterization. Starting from DNA extraction of the intestinal microbial community, the subsequent high‐throughput sequencing provides taxonomic insight into the gut microbiota down to the genus and species levels for 16S rRNA gene and ITS microbial profiling respectively. In addition to the taxonomic composition, whole shotgun metagenomics allows the reconstruction of the microbial genomes and prediction of the bacterial functional features.

Metagenomics allows the assessment of the composition, encoded activities and functional features of the entire intestinal microbial community, including bacterial taxa that have not yet been cultivated under *in vitro*, i.e. laboratory conditions, as well as the detection of non‐bacterial taxa that colonize the GIT such as protozoa, fungi, viruses and archaea (Hamady and Knight, 2009; Ventura *et al*., 2009; Blake and Suchodolski, 2016; Fig. 1). Historically, 16S rRNA gene‐based microbial profiling was the first culture‐independent method to be employed for the compositional profiling of the gut microbiota. This approach employs universal primers for PCR‐mediated amplification and subsequent sequencing of a single or multiple hypervariable regions of the 16S rRNA gene, which represents a conserved phylogenetic marker composed by highly conserved sequences interspersed with nine hypervariable regions (Neefs *et al*., 1993; Hamady and Knight, 2009; Milani *et al*., 2017a, 2017b,2017a, 2017b). However, despite the fact that the 16S rRNA microbial profiling method was and continues to be the most cost‐effective and popular technique to study gut microbiota composition, it has a number of serious limitations. There is currently no single standardized methodology for DNA extraction, nor is there agreement among the scientific community which primers are most appropriate for amplification or which hypervariable region of the 16S rRNA gene is to be targeted to achieve optimal sequencing efficiency of a DNA region with the highest level of taxonomic discriminatory power (Mancabelli *et al*., 2020). Furthermore, 16S rRNA gene‐based microbial profiling generates bacterial taxonomic composition data typically down to the genus level only, while it commonly fails in detecting underrepresented bacterial taxa, thereby causing biases in the interpretation and comparison of data from different studies (Chakravorty *et al*., 2007). More recently, the so‐called internal transcribed spacer (ITS) microbial profiling methods were introduced as a tool to offer a more refined taxonomic view of the gut microbiota (Milani *et al*., 2014, 2018). The ITS region is located between the 16S rRNA and 23S rRNA genes within the rRNA locus. This genomic portion is more variable at the interspecies level when compared to the 16S rRNA gene, and represents a more suitable phylogenetic marker to obtain an in‐depth view of the intestinal microbial population by providing an accurate species‐ or even subspecies‐level taxonomic resolution (Milani *et al*., 2017a, 2017b,2017a, 2017b). However, this method is currently only available for specific microbial genera such as *Bifidobacterium* and *Lactobacillus* (Milani *et al*., 2014, 2018).

In‐depth whole metagenome shotgun sequencing (WMS), which involves high‐resolution sequencing of total microbial DNA extracted from a specific matrix, captures substantially more information when compared to gene or sequence‐specific amplification‐based approaches. Indeed, in addition to taxonomic composition, WMS offers in‐depth insights into the genomic content and compositional arrangement of bacterial consortia, also allowing predictions of their functional features (Lugli *et al*., 2019). Notably, functional classification of WMS data may be used to shed light on the microbial dark matter in order to identify the catabolic and anabolic activities of intestinal bacteria and to investigate the presence of genes involved in multiple processes, such as adhesion to the intestinal epithelium or antibiotic resistance (Rinke *et al*., 2013). Furthermore, WMS is not affected by the potential bias caused by the amplification step typical of the 16S rRNA or ITS microbial profiling methods. Despite these advantages, many of the large‐scale studies continue to involve 16S rRNA microbial profiling because of the high costs of high‐resolution WMS. In order to overcome the limitations of the 16S rRNA or ITS microbial profiling, shallow shotgun sequencing, which is a low‐depth WMS, has recently been proposed to combine species‐level taxonomic assignment with reduced cost while avoiding amplification bias of 16S rRNA‐based sequencing (Hillmann *et al*., 2018). However, regardless of the method used, metagenomics is a ‘relative approach’ (Vandeputte *et al*., 2017). Indeed, it provides a microbial profiling based on relative and not absolute abundance, thus hampering efforts to correlate microbiome features to quantitative data, including physiological parameters or metabolite levels (Vandeputte *et al*., 2017). Evaluation of the overall microbial abundance through quantitative approaches such as flow cytometry has recently been proposed to overcome this limitation so as to provide quantitative microbiome profiling (Vandeputte *et al*., 2017).

In addition to metagenomics, further ‘omics’ approaches, in particular metatranscriptomics and metabolomics, have been developed in order to move beyond mere taxonomic assignment or gene functional predictions. Indeed, metatranscriptomics identifies which genes, among the myriad of bacterial genes that constitute the gut microbiome, are actually transcribed through RNA sequencing of a given sample. In addition, metabolomics provides information on the production of microbial metabolites, thereby attempting to capture metabolic activity among and interactions between the gut microbiota and its host (Smirnov *et al*., 2016; Wang *et al*., 2020).

## Insights into the gut microbiota of healthy dogs and cats

Combining culture‐dependent approaches with metagenomic methodologies for studying the canine and feline gut microbiota has shown that bacterial abundance and biodiversity gradually increase along the GIT (Ritchie *et al*., 2008; Suchodolski *et al*., 2008; Honneffer *et al*., 2017). Specifically, bacterial cultivation efforts revealed that the total microbial load of the stomach and small intestine of dogs and cats is lower than that found in the distal intestinal tract, with an overall bacterial abundance along the GIT that ranges from 10^2^ up to 10^14^ colony‐forming units (CFU) per gram of luminal content (German *et al*., 2003; Mentula *et al*., 2005; Ritchie *et al*., 2008). Furthermore, the small intestine is inhabited by both aerobic and facultative anaerobic bacteria, while the large intestine is mostly colonized by anaerobic microbes, reflecting the microenvironment and oxygen availability of these different GIT compartments. Furthermore, cultivation‐independent molecular methods revealed variations of microbial richness along the length of the intestinal tract, with the greatest biodiversity recorded in the large intestine when compared to the stomach and small intestine. This observation is in accordance with the physiological functions of these different intestinal compartments, with the colon and caecum as the main fermentative sites in monogastric mammals (Suchodolski *et al*., 2008; Suchodolski, 2016; Honneffer *et al*., 2017). Despite the well‐accepted notion that variations in bacterial abundance, taxonomic composition and biodiversity occur along the GIT, much of the relevant published scientific literature bases its gut microbiota findings on the analysis of faecal samples due to practical difficulties and ethical constraints related to the collection of samples from each intestinal sector. However, although starting from the same environmental matrix (i.e. stool samples), comparison of the results obtained by different studies has revealed distinct differences between the bacterial populations present in the GIT of cats and dogs. In this context, multiple factors, including diet, environment, age, gender, genetics, diseases and relative therapies, have been shown to affect the intestinal microbial composition of an individual, promoting interindividual fluctuations among animals of the same species with each pet apparently possessing its own unique microbial gut ecosystem (David *et al*., 2014; Wernimont *et al*., 2020). Furthermore, non‐standard experimental procedures applied by different metagenomics‐based studies, due to different DNA extraction protocols, distinct primers employed for amplification of 16S rRNA gene‐associated hypervariable regions and/or varying bioinformatics methodologies for the interpretation and analysis of metagenomic data, have contributed to poor reproducibility across populations (Schloss, 2018). Despite this limitation in performing a straightforward comparison between findings of different studies, key bacterial players have transversely been found in feline and canine faeces in different studies, regardless of the metagenomic approaches used. Specifically, Fusobacteria, Bacteroidetes and Firmicutes were identified as the predominant and prevalent bacterial phyla characterizing the faecal microbiota of dogs and cats (Ritchie *et al*., 2010; Handl *et al*., 2011; Minamoto *et al*., 2012; Tun *et al*., 2012; Alessandri *et al*., 2019a, 2019b, 2019c). Within the Firmicutes phylum, Bacilli, Clostridia and Erysipelotrichi are the most representative bacterial classes for both canine and feline gut microbiota. Specifically, the Bacilli class is predominantly represented by *Streptococcus* and *Lactobacillus* genera in dogs and *Enterococcus* and *Lactobacillus* in cats (Handl *et al*., 2011). The Clostridia class is represented by *Clostridium* clusters IV (*Ruminococcaceae* family and *Faecalibacterium prausnitzii*), XI (*Peptostreptococcaceae* family) and XIVa (*Lachnospiraceae* family and *Blautia*), while the Erysipelotrichi is mainly represented by *Turicibacter*, *Catenibacterium* and *Coprobacillus* genera (Ritchie *et al*., 2010; Handl *et al*., 2011; Pilla and Suchodolski, 2019). Furthermore, *Prevotella* and *Bacteroides*, belonging to the Bacteroidetes phylum, are among the most abundant and prevalent bacterial genera of the faecal microbiota of both companion animals considered here (Alessandri *et al*., 2019a, 2019b, 2019c, 2020).

Furthermore, *Fusobacterium*, a genus belonging to the Fusobacteria phylum, was identified as one of the principal microbial players of the canine and feline gut microbiota, though with a lower average relative abundance in cats (Suchodolski *et al*., 2008; Alessandri *et al*., 2019a, 2019b, 2019c, 2020). Notably, in humans, *Fusobacterium* is associated with inflammatory bowel diseases, while the presence of a specific species of this genus in the human intestine, i.e. *Fusobacterium nucleatum*, is linked to the occurrence of colorectal cancer due to its potential carcinogenic features (Ng *et al*., 2019; Gethings‐Behncke *et al*., 2020). However, this negative association has not been observed for dogs and cats. Indeed, *Fusobacterium* has been found to be present at high abundance in the gut microbiota of healthy dogs and cats, and its common presence in the GIT of other carnivorous animals suggests that it is not harmful to its host (Ley *et al*., 2008; Swanson *et al*., 2011; Alessandri *et al*., 2019a, 2019b, 2019c, 2020). The ability of *Fusobacterium* species to degrade proteins to obtain their preferred growth substrates, i.e. amino acids and peptides, is probably the reason for its high abundance in carnivorous animals (Doron *et al*., 2014). Furthermore, not all members of the *Fusobacterium* genus should be considered as potentially harmful to the host. For example, it has been demonstrated that *Fusobacterium varium* not only acts as a potent antagonist of pathogen colonization, but is also able to exert anti‐inflammatory effects and sustain enterocytes by producing butyrate from protein fermentation (Potrykus *et al*., 2008). Indeed, while most of intestinal butyrate is obtained through bacterial fermentation of dietary fibres via two metabolic pathways, one involving the phosphorylation of the butyryl‐CoA to synthetize butyryl‐phosphate that is then transformed into butyrate via butyrate kinase and the other including the transfer of the CoA moiety of butyryl‐CoA to acetate leading to the production of butyrate and acetyl‐CoA, metagenomic data analyses revealed that butyrate can be also produced from proteins via the lysine pathways (Vital *et al*., 2014; Louis and Flint, 2017; Liu *et al*., 2018). In this context, specific ecological studies coupled with comparative genomic analyses are required to shed light on the significance of the high abundance of *Fusobacterium* and its role in the gut microbiota of dogs and cats.

With a reduced abundance but an equivalent prevalence of the co‐dominant Bacteroidetes, Firmicutes and Fusobacteria, the Proteobacteria and Actinobacteria phyla represent two other major taxa of the canine and feline gut microbiota (Tun *et al*., 2012; Alessandri *et al*., 2019a, 2019b, 2019c). Specifically, Proteobacteria are more abundant in dogs, while the relative abundance of Actinobacteria is higher in the feline faecal microbiota (Handl *et al*., 2011; Moon *et al*., 2018). Among the Proteobacteria, *Sutterella*, *Succinivibrio*, *Anaerobiospirillum* and *Escherichia/Shigella* are the most abundant genera in both canine and feline faeces (Handl *et al*., 2011). In this context, *Escherichia*/*Shigella* together with another genus of the Proteobacteria phylum, i.e. *Salmonella*, which is also a member of the *Enterobacteriaceae*, are generally considered gastrointestinal pathogens of public concern (Moon *et al*., 2018). However, most strains belonging to the abovementioned bacterial genera are non‐pathogenic and indeed contribute to the microbiome function of healthy hosts (Moon *et al*., 2018). Notably, a metagenomic study revealed that the *Coriobacteriaceae* family is the main representative of the Actinobacteria phylum, with *Collinsella* and *Slackia* as dominant genera in dogs, while *Eggerthella* and especially *Olsenella* being prevalent in cats (Handl *et al*., 2011). Furthermore, recently, the application of the bifidobacterial ITS microbial profiling method to multiple canine and feline faecal samples revealed the presence of several *Bifidobacterium* species in each processed sample (Alessandri *et al*., 2019a, 2019b, 2019c, 2020), thus strengthening the notion that bifidobacteria are ubiquitous commensal microorganisms of the intestinal microbial community of mammals, including dogs and cats (Milani *et al*., 2017a, 2017b,2017a, 2017b).

**Fig. 2:**
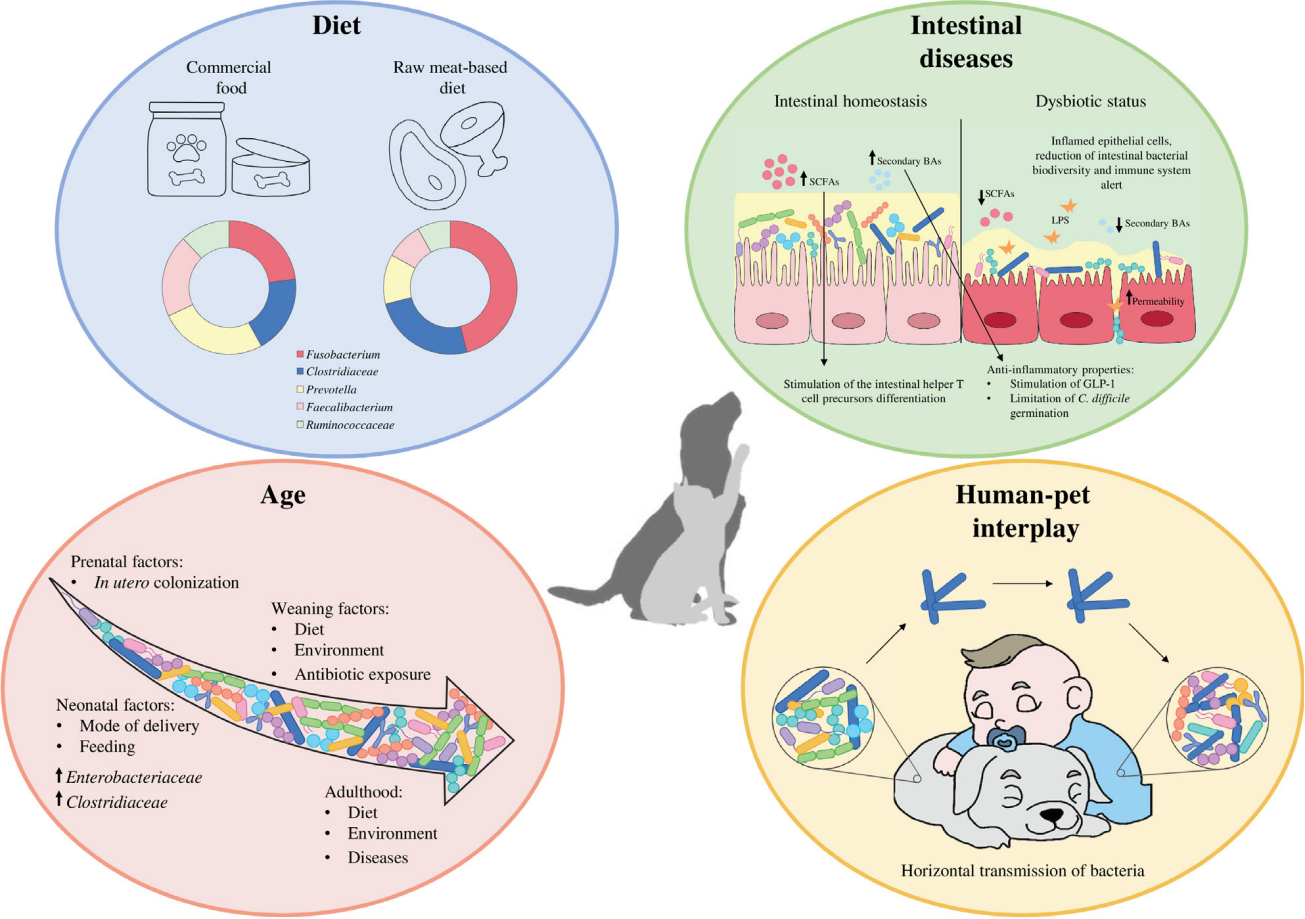
Schematic representation of the main factors influencing the canine and feline gut microbiota. Specifically, age, diet, perturbation of the gut microbiota homeostasis and human–pet interplay play a crucial role in the modulation of the intestinal microbial community of dogs and cats.

Although key bacterial members are commonly present in the faecal microbial community of healthy dogs and cats, a finding that is indicative of a core faecal microbiota, the taxonomic composition may be subject to changes and modulations due to the influence of several factors including diet (Bresciani *et al*., 2018; Schmidt *et al*., 2018; Alessandri *et al*., 2019a, 2019b, 2019c), age (Fahey *et al*., 2008; Masuoka *et al*., 2017), metabolic disorders (i.e. diabetes or obesity; Alexander *et al*., 2018; Salas‐Mani *et al*., 2018), intestinal dysbiosis (diarrhoea or inflammatory bowel diseases) or cancer (Honneffer *et al*., 2014; Omori *et al*., 2017; Kalenyak *et al*., 2018), as well as anthropogenic influences (Alessandri *et al*., 2019a, 2019b, 2019c; Fig. 2).

## The effect of changes in dietary habits on canine and feline gut microbiota

Diet has been recognized as one of the main drivers influencing both biodiversity and functional features of the mammalian gut microbiota (Ley *et al*., 2008; David *et al*., 2014). Indeed, nutrients introduced through the diet not only act as sustenance for the host, but diet components that cannot be directly digested by the host may also represent nutrients for gut microorganisms.

### Inclusion of prebiotics in canine and feline diet

Despite its original carnivorous classification, the domestic dog is currently considered as an omnivorous animal, since commercial pet foods are formulated to provide a balanced nutritional intake yet with the supplementation of high concentrations of fibre and carbohydrates (generally higher than 3% and 30%, respectively; Barry *et al*., 2012; Alessandri *et al*., 2019a, 2019b, 2019c), which is necessary to produce extruded dry diet (kibble) (Pilla and Suchodolski, 2019). The domestic cat, on the other hand, with its short colon represents an example of an evolutionary adaptation to a strict carnivorous diet and is still considered an obligate carnivore. Nonetheless, commercial feeds for domesticated felines are enriched in components of vegetable origin (Hooda *et al*., 2013; Rochus *et al*., 2014; Butowski *et al*., 2019). Furthermore, in recent years and based on a multitude of studies that have highlighted health‐promoting features of prebiotics, several companies have started to include prebiotic compounds in commercial pet foods (Deng and Swanson, 2015). Prebiotics are defined as selectively fermented ingredients that are able to induce specific changes in the composition and/or functional activities of the intestinal bacterial community, in order to confer benefits to the host (Gibson *et al*., 2004). Several non‐digestible carbohydrates are known to possess prebiotic features and range from the disaccharide lactulose to oligo‐ or polysaccharides such as fructo‐oligosaccharides (FOS), mannan‐oligosaccharides (MOS), xylo‐oligosaccharides (XOS) and galacto‐oligosaccharides (GOS) and inulin (Roberfroid *et al*., 2010; Slavin, 2013). To be effective, prebiotics have to withstand digestion by host enzymes and reach the distal part of the intestine where they favour the proliferation and metabolic activities of specific bacterial species able to promote evident beneficial effects to the host (Pinna and Biagi, 2016). By escaping upper intestinal hydrolysis and absorption and reaching the hindgut compartments, these complex carbohydrates will be selectively fermented by those microorganisms that possess genes encoding specific extracellular and/or intracellular glycosyl‐hydrolases (GH) and transport systems, required for the breakdown and uptake, respectively, of these carbohydrates (Vieira *et al*., 2013). The main products of this fermentative catabolic process are SCFAs, which include saturated aliphatic organic acids, mainly represented by butyrate, acetate and propionate. These particular organic acids are believed to improve host health by increasing mineral absorption, regulating bowel functions, decreasing the luminal pH thus limiting the proliferation of pH‐sensitive pathogenic bacteria, influencing the immune system and providing nutrients to enterocytes (Ratajczak *et al*., 2019). In this context, several studies have highlighted the beneficial effects of prebiotic inclusion in the diet of dogs and cats (Anderson *et al*., 1991; Barry *et al*., 2010; Nogueira *et al*., 2019; Ide *et al*., 2020).

A recent study assessed the impact of supplementation of 7.5% beet pulp, which is a commonly used dietary fibre for the formulation of commercial canine feed, consisting of a mixture of fermentable and non‐fermentable carbohydrates (Middelbos *et al*., 2010). This trial revealed a drastic shift of the Firmicutes/Fusobacteria ratio in favour of the former in the faecal microbiota of treated dogs, accompanied by a threefold increase of *Faecalibacterium prausnitzii* and proliferation of *Eubacterium hallii* (Middelbos *et al*., 2010). Another study was aimed at evaluating the impact of kestose (a type of FOS) supplementation on the canine gut microbiota and SCFA concentrations. While the *Lactobacillus* genus was shown to elicit a slight and statistically non‐significant increase in relative abundance, the *Bifidobacterium* taxa showed a marked trend of numerical enhancement during the eight weeks of treatment, thus supporting the bifidogenic effect of this specific FOS component. Furthermore, based on the observation that kestose was shown to cause a decreased relative abundance of *Clostridium perfringens* and a simultaneous increase of butyrate concentration, it was inferred that the increased concentration of butyrate lowers luminal pH, thus creating a less suitable environment for the proliferation of certain microbial species (Ide *et al*., 2020). Moreover, the administration of a FOS (4%)‐ or pectin (4%)‐based diet to cats was shown to cause an increase in the relative abundance of the *Bifidobacterium* genus and *Lactobacillus* genus, respectively, when compared to the control diet (supplemented with 4% cellulose). Furthermore, the total concentration of SCFAs was higher in faecal samples of treated cats when compared to the cellulose control (Barry *et al*., 2010). Taken together, these results highlight the important role of diet in modulating the intestinal bacterial community as well as its functional activities in both dogs and cats.

### Raw meat‐based diet

Despite extensive efforts dedicated to the formulation of industrial pet food in order to guarantee a balanced diet as well as the inclusion of prebiotics to promote intestinal homeostasis, an apparently opposing trend has recently emerged. Indeed, it has become increasingly popular to feed dogs and cats with a raw meat‐based diet (RMBD), instead of the more conventional commercial dry food (van Bree *et al*., 2018). The choice of many pet owners to return to a ‘natural’ and ‘home‐made’ diet for their animals was driven by several factors. First, most industrial foods are obtained from the use of waste and/or by‐products from human‐oriented food processing industry, thus failing in formulating products of first choice. Furthermore, while the extruded and retort format, which is typical of commercial food, uses heat, moisture and pressure to process food, the RMB diet incorporates food that is essentially unprocessed (Algya *et al*., 2018). Second, pet owners can influence health and well‐being of their companion animals through the feed they provide to their pets (Schlesinger and Joffe, 2011). As the RMBD designation suggests, these formulations include raw meat, internal organs, cartilage, fat and fleshy bones from farm animals (ruminants, poultry and pigs), horses, game and fish (Fredriksson‐Ahomaa *et al*., 2017). Furthermore, though at a much reduced level when compared to the extruded diet, RMBD can also contain vegetables, eggs and dairy products (van Bree *et al*., 2018). The so‐called BARF (Bones and Raw Food or Biologically Appropriate Raw Food) diet is probably the most widespread and known example of RMBD. In parallel with the take‐up of these novel nutritional approaches, the advantages and disadvantages of feeding a natural diet were investigated. While it has been demonstrated that RMBD provides important health benefits to its animal consumer, including reduction in dental diseases coupled with fresher breath, alleviation of arthritis, increase of the immune response, healthier skin and a shiny coat, several issues of public concern were raised (Freeman *et al*., 2013). Indeed, some studies reported findings that discourage the administration of this natural diet since it does not guarantee a balanced nutritional intake and it increases the risk of exposure to zoonotic pathogens, encompassing *Campylobacter* spp., *Salmonella* spp., *Yersinia* spp. and pathogenic *Escherichia coli* strains, thus threatening the health of both animal companions and their owners (Schlesinger and Joffe, 2011; Kim *et al*., 2017; van Bree *et al*., 2018). However, these studies refer only to nutritional and pathogen contents, while they do not take into account any possible changes both in microbial composition and in functional activities of the gut microbiota of dogs and cats when they are fed on a RMB diet. Therefore, these investigations do not fully describe the advantages/disadvantages of this natural diet and care should be taken to draw far‐reaching conclusions from such findings.

Collectively, even if the studies investigating the impact of a RMBD on the gut microbiota of dogs and cats are based on different experimental design and sequencing protocols, they share common features (Table 1). In general, the BARF diet seems to favour the proliferation of the *Fusobacterium* genus and *Clostridiaceae* family. As already mentioned, members of the *Fusobacterium* genus were pointed out as potentially harmful microorganisms, being related to intestinal bowel diseases and colorectal carcinoma in humans (Castellarin *et al*., 2012; Kostic *et al*., 2013; Alhinai *et al*., 2019). In the same way, *Clostridiaceae* is a highly diverse family, including not only useful bacterial genera involved in nutrient digestibility, yet also pathogenic microorganisms, i.e. *Clostridium difficile* and *Clostridium perfringens* (Rajilic‐Stojanovic and de Vos, 2014). Based on these notions, it seems that the BARF diet undermines canine and feline health. However, by definition, these opportunistic pathogens are not always harmful to the host, but only in case of perturbations of the bowel homeostasis. Under normal circumstances, such bacteria behave like commensal microorganisms in the intricate microbial ecosystem of the host’s intestine. In this context, a positive correlation appears to exist between *Clostridiaceae* and protein digestibility and protein dietary content, suggesting that members of this microbial family are involved in protein digestion in the GIT of dogs or that they can thrive in a carbohydrate‐poor environment (Bermingham *et al*., 2017). Similarly, *Clostridiaceae* were shown to positively correlate with faecal health scores (measured through firmness of faecal material), while a negative correlation was observed with faecal output, indicating that the increased abundance of *Clostridiaceae* taxa in the faecal microbiota of dogs fed on a BARF diet is not detrimental for canine health, but, rather, is associated with protein degradation (Bermingham *et al*., 2017). To further confirm the involvement of this taxon in protein fermentation, various *Clostridium* species, in particular *Clostridium ramosum*, *Clostridium rectum*, *Clostridium hiranonis* and *Clostridium perfringens*, were detected at a significantly higher relative abundance level in faecal samples of healthy dogs fed on a natural diet when compared to the control group (Kim *et al*., 2017). In addition, it has recently been demonstrated that *Clostridium perfringens* is, in carnivores, responsible for the generation of butyrate from protein due to the presence, in its genetic repertoire, of a butyrate kinase‐encoding gene involved in the main butyrate‐synthesis pathway (Vital *et al*., 2015). Similarly, as mentioned in the previous paragraph, also some members of the *Fusobacterium* genus are able to produce butyrate via amino acid fermentation, starting from protein degradation (Potrykus *et al*., 2008). Therefore, the ability of proteolytic microorganisms to produce SCFAs may explain why only the faecal organic acid profiles were shown to be affected by diet, but not the total level of SCFAs, in either dogs or cats (Sandri *et al*., 2017; Butowski *et al*., 2019).

**Table 1:** Effects of feeding a raw meat‐based diet on canine and feline gut microbiota.

Animal species	Effect of the raw meat‐based diet	References
Dog	Increase of the Fusobacteria and Proteobacteria phyla. Decrease of Firmicutes, especially of *Ruminococcaceae* and *Erysipelotrichaceae* family and the *Faecalibacterium* genus	Schmidt *et al*. (2018)
Dog	Diet affected 27 microbial families and 53 genera. *Bacteroides*, *Prevotella*, *Peptostreptococcus* and *Faecalibacterium* were reduced while *Fusobacterium*, *Collinsella*, *Slakia Lactobacillus* and *Clostridium* increased.	Bermingham *et al*. (2017)
Dog	Decreased proportion of *Lactobacillus*, *Paralactobacillus* and *Prevotella* with a parallel higher relative abundance of *Clostridium*, *Bacteroides* and *Fusobacterium*.	Sandri *et al*. (2017)
Dog	Decreased abundance of *Prevotella*, *Faecalibacterium* and *Sutterella* coupled with reduced GH families involved in the degradation of complex plant‐derived polysaccharides. Higher abundance of Fusobacteria and Actinobacteria and increased proportion of genes related to amino acid and fatty acid/lipid degradation pathways.	Alessandri *et al*. (2019)
Cat	Higher abundance of *Fusobacterium*, *Eubacterium* and *Clostridium* together with a reduced abundance of *Prevotella*	Butowski *et al*. (2019)
Cat	Greater proportions of *Peptococcus*, *Pseudobutyrivibrio* and significantly reduced abundance of *Faecalibacterium* and *Succinivibrio*. Higher abundance, yet not statistically significant of *Fusobacterium* and *Clostridium*.	Kerr *et al*. (2014)

At the same time and in parallel with the numerical enhancement of proteolytic bacteria, a significant reduction was observed in the abundance of saccharolytic microorganisms, such as *Prevotella* and *Faecalibacterium* genera together with members of the *Ruminococcaceae* family (Herstad *et al*., 2017; Alessandri *et al*., 2019a, 2019b, 2019c). Notably, these taxa are generally associated with high‐fibre and carbohydrate intake since they are able to metabolize a wide variety of dietary glycans (David *et al*., 2014). In addition, the observation that differences in the proportion of macronutrients in the canine and feline diet cause gut microbiota compositional changes was confirmed from a functional point of view. Indeed, a shotgun metagenomics analysis involving a faecal sample per group of diet (commercial diet vs. BARF) revealed a higher abundance of GH families involved in the breakdown of complex plant‐derived polysaccharides being associated with a commercial food diet, while a greater proportion of genes involved in amino acid degradation and fatty acid/lipid degradation pathways was observed in the faecal samples from dog fed on a diet of raw meat (Alessandri *et al*., 2019a, 2019b, 2019c). In parallel, the RMBD was shown to play a role in modulating the intestinal metabolome as a BARF‐based diet causes an increase in faecal cholesterol and other metabolites, such as isomaltose, the inhibitory neurotransmitter gamma‐aminobutyric acid (GABA) and its precursor gamma‐hydroxybutyric acid (GHB; Schmidt *et al*., 2018).

Taken together, these changes emphasize the plasticity of the gut microbiota in adapting to different dietary components.

## Evolution of the canine and feline intestinal community during their life span

For a long time, it was thought that development of the mammalian gut microbiota occurs immediately after birth. However, in recent years, the dogma of a sterile *in utero* environment has been challenged by different studies that have highlighted the existence, in humans and rat, of an intrauterine and placenta microbial community in healthy full‐term pregnancies (Aagaard *et al*., 2014; Perez‐Munoz *et al*., 2017; Mancino *et al*., 2019). Despite these publications, the assumption that the very first bacterial contamination of the gut microbiota may occur before birth is still highly controversial (Jimenez *et al*., 2008; Walker *et al*., 2017; Kuperman *et al*., 2019; Rackaityte *et al*., 2020). Instead, it is well accepted that the earliest colonizers of the mammalian GIT, including dogs and cats, are generally represented by facultative anaerobes, especially members of the *Enterobacteriaceae* family, which are believed to be responsible for the elimination of oxygen present in the gastrointestinal tract immediately following birth (Matamoros *et al*., 2013; Moon *et al*., 2018). This oxygen depletion event transforms the intestinal tract into a suitable environment for strictly anaerobic microorganisms (Moon *et al*., 2018). Substantial variations in the total bacterial load as well as in the taxonomic composition of the gut microbiota of newborns may occur depending on multiple factors, including mode of delivery (natural vs. C‐section delivery), type of feeding (breast or formula milk) and antibiotic exposure (Fugelli and Laerum, 1989; Del Chierico *et al*., 2015; Imoto *et al*., 2018). For example, it has been demonstrated that the gut microbiota of vaginally delivered infants, which have been in contact with the maternal vaginal and faecal microbiota, is generally colonized by mother‐to‐infant vertically transmitted microorganisms. Conversely, Caesarean section delivered infants are not directly exposed to maternal bacteria, and their intestinal tract is for this reason mainly colonized by skin‐ and environment‐associated microorganisms (Dominguez‐Bello *et al*., 2010; Backhed *et al*., 2015). Nevertheless, to date, no studies have attempted to evaluate the impact of delivery mode and/or feeding type on the canine and feline gut microbiota due to the difficulties in collecting faecal samples from pups and milk form dams. However, an ecological survey investigating the bifidobacterial population harboured by the intestinal tract of multiple mammals revealed the presence of shared bifidobacterial phylotypes among the faecal samples of mother–offspring dyads, including canine and feline dams with their respective puppies and kittens (Milani *et al*., 2017a, 2017b,2017a, 2017b). Furthermore, siblings from the same litter were shown to harbour a more similar bifidobacterial population when compared to pups from different litters (Milani *et al*., 2017a, 2017b,2017a, 2017b). These findings therefore support the notion that, from humans to other mammals, and including companion animals, vertical transmission events from mother to her offspring play a key role in defining the biodiversity and composition of the newborn gut microbiota. Furthermore, these results corroborate the current view that members of the *Bifidobacterium* genus are among the first colonizers of the intestinal tract of various animals that provide parental care to their offspring (Bunesova *et al*., 2014). However, in humans the relative abundance of this genus gradually declines during ageing, declining from the first years of life, adolescence and adulthood, to old age (Arboleya *et al*., 2016). A similar trend was observed for dogs and cats. Indeed, several studies reported a higher abundance of the *Bifidobacterium* taxa in puppies and kittens during the lactation and post‐weaning phases when compared to adult cats and dogs (Jia *et al*., 2011; Guard *et al*., 2017; Alessandri *et al*., 2019a, 2019b, 2019c).

Beyond the mode of delivery, feeding type represents another major factor that influences the early microbial colonization, thus playing a role in the modulation of the neonatal intestinal microbial population and relative functions. Indeed, mammalian milk not only acts as a vector for certain bacteria to transfer from mother to offspring, but it also provides a mix of nutrients as well as antimicrobial agents and immunoglobulins (Martin *et al*., 2003; O'Sullivan *et al*., 2015; Chastant‐Maillard *et al*., 2017; Milani *et al*., 2017a, 2017b,2017a, 2017b). In this context, it has been reported that canine milk is a natural source of *Lactobacillus* species that can be transmitted to suckling puppies (Martin *et al*., 2010). In parallel, a high abundance of lactobacilli was observed in the faeces and luminal content of puppies (Buddington, 2003; Vilson *et al*., 2018). Therefore, it is possible that canine milk, similarly to human milk, acts as a carrier of microorganisms that are then able to colonize the intestinal tract of pups. Certainly, studies aimed at evaluating the presence of shared *Lactobacillus* strains in milk and faecal samples of puppy or kitten and their corresponding canine or feline parents are required to validate this hypothesis.

Weaning marks another important step in the establishment and development of the host’s intestinal microbial community. As already observed in humans, the transition from a liquid to a solid and more diverse diet (around the fourth/fifth week of life in dogs and cats) is, indeed, generally accompanied by an increase in the biodiversity of the pups' gut microbiota (Backhed *et al*., 2015; Guard *et al*., 2017). In this context, a longitudinally study demonstrated that while Fusobacteria and Proteobacteria maintain similar percentages at all assessed time points (2, 21, 42 and 56 days of life), the Firmicutes phylum dominated the intestinal microbial community at 2 days of age, while Bacteroidetes was underrepresented to reach a higher relative abundance typical of an adult intestinal gut community starting from day 21 (Guard *et al*., 2017). Specifically, *Lactobacillus*, *Clostridium* spp. and *Escherichia coli* were prominent in the first days after birth to gradually decrease over time to give way to other multiple bacteria for intestinal colonization (Buddington, 2003; Guard *et al*., 2017). In a similar way, a culture‐based analysis revealed that *Enterobacteriaceae* and *Enterococcus* spp. dominated the gut microbiota of pre‐weanling kittens, while their abundance decreased throughout life (Masuoka *et al*., 2017). In addition, as observed in dogs, also the gut microbiota of lactating kittens is characterized by high numbers of *Clostridium* spp. but a low relative abundance of *Bacteroides* which, instead, will increase during adult life (Fahey *et al*., 2008). Although weaning causes profound changes in the gut microbiota of puppies and kittens with a bacterial community that begins to acquire the stability typical of the adult life, further changes may occur. Indeed, it has been demonstrated that eight weeks after birth the faecal microbial population of puppies is still significantly different from that of their mother, and by extension from that of adult dogs (Guard *et al*., 2017). Similarly, a shotgun metagenomic‐based longitudinal study of feline faecal samples revealed significant differences in both functional activities and taxonomic composition at 18 weeks of age when compared to the 30 week age point (Deusch *et al*., 2015). Despite the lack of long‐term studies focusing on the gut microbiota of dogs and cats during life, it is well accepted that following the evolutions of the first months of life, as it has been shown for humans, the canine and feline intestinal microbial community remains stable throughout adulthood to further changes during old age (O'Toole and Jeffery, 2015; Pilla and Suchodolski, 2019). In this context, human senescence is generally associated with consistent alterations in nutrition, lifestyle and physiology accompanied by a decline of the immune system (immunosenescence) resulting in a chronic low grade of inflammation with consequences on the intestinal microbial community (Candela *et al*., 2014; O'Toole and Jeffery, 2015). Indeed, ageing usually correlates with higher levels of gut oxygen concentration and reactive oxygen microbial species, thus inactivating strict anaerobes and compromising the overall functionality of the intestinal population, ultimately resulting in a reduced microbial biodiversity and pauperization of genes involved in SCFA production (Candela *et al*., 2014). In this context, age is, next to diet, an important factor affecting the gut microbiota composition of dogs and cats, which is consistent with similar observations in humans.

## Metabolic disorders and alterations of the canine and feline gut microbiota

During recent decades, obesity and overweight have rapidly increased in pet populations, which has caused a major concern for companion animal health (Grzeskowiak *et al*., 2015). An imbalance between energy intake and energy expenditure is the main cause of obesity. However, additional factors contribute to the onset of this metabolic disorder. Indeed, as a consequence of domestication, dogs and cats have started to experience a sedentary life with reduced physical activity and *ad libitum* access to high‐caloric diet which, coupled with genetic predisposition and neutering, represent the basis of the multifactorial aetiology of obesity (Grzeskowiak *et al*., 2015; Osto and Lutz, 2015; Tal *et al*., 2020). In addition, advances in microbiome studies have demonstrated that gut microbiota plays a role in extra‐intestinal disorders, including obesity and diabetes (Park *et al*., 2015; Blake and Suchodolski, 2016; Forster *et al*., 2018, Chun *et al*., 2020). In this context, the relative abundance of the Actinobacteria and Fusobacteria phyla and *Roseburia* genus increased in obese dogs when compared to the lean group (Kieler *et al*., 2017; Chun *et al*., 2020). Specifically, among the Fusobacteria phylum, *Fusobacterium mortiferum* and *Fusobacterium perfoetens* were positively correlated with an overweight status, thus implying a contribution of these two bacterial species to canine obesity (Chun *et al*., 2020). Conversely, a general decrease of bacterial taxa involved in SCFA production was observed in obese dogs, including the *Blautia* and *Eubacterium* genera and *Lachnospiraceae* family (Handl *et al*., 2013; Forster *et al*., 2018). Furthermore, a parallel reduced abundance of carboxylic acids, encompassing linoleic acid, ferulic acid and colnelenic acid was registered in obese dogs (Forster *et al*., 2018). Since these metabolites act as antioxidants as well as anti‐inflammatory compounds, their decreased abundance may reflect the subclinical chronic inflammation and oxidative stress typical of an overweight condition (Forster *et al*., 2018). In obese cats, instead, Fusobacteria as well as Bacteroidetes and *Clostridium* cluster XIVa showed reduced abundance when compared to the lean group, while *Enterobacteriaceae* displayed an opposite trend (Kieler *et al*., 2016). A diet intervention based on monitoring calorie intake is the elective therapy for treating canine and feline obesity. Furthermore, inclusion of dietary prebiotics seems to aid in promoting health in overweight or obese dogs and cats (Li *et al*., 2017; Alexander *et al*., 2018; Apper *et al*., 2020). In this context, the administration of short‐chain fructo‐oligosaccharides to obese dogs was shown to impact on gut microbiota composition and fermentative activity coupled with an increased microbial biodiversity, which is considered a positive effect (Apper *et al*., 2020). Specifically, an increase in SCFA bacterial producers, i.e. *Roseburia* and *Blautia,* was observed in parallel to an increment in faecal butyrate concentration that not only acts as an anti‐inflammatory molecule decreasing intestinal permeability, but also regulates levels of anti‐inflammatory gut hormones such as the glucagon‐like peptide 1 (GLP‐1) responsible for enhancing glucose‐dependent insulin secretion by pancreatic β‐cells (Apper *et al*., 2020). Similarly, another study reported the increased abundance of butyrate in the faeces of obese dogs supplemented with inulin‐type fructans, as well as an increase in the relative abundance of *Eubacterium* and *Turibicater*, while the Proteobacteria phylum showed an opposite trend (Alexander *et al*., 2018).

Furthermore, an overweight condition is generally associated with several other comorbidities such as diabetes mellitus (DM), osteoarthritis or cardiovascular diseases that undermine canine and feline health (Grzeskowiak *et al*., 2015; Phillips *et al*., 2017). In this context, while dogs generally display type 1 DM, cats are more likely to be affected by type 2 DM (Wernimont *et al*., 2020). Similarly to obesity, altered gut microbiota composition has been related to diabetes (Blake and Suchodolski, 2016). Specifically, dogs with DM are characterized by both intestinal dysbiosis with higher proportion of *Enterobacteriaceae* and alterations of the faecal concentration of unconjugated bile acids (Jergens *et al*., 2019). In the same way, a decreased gut microbial biodiversity and a drastic reduction of butyrate‐producing bacteria occur in cats affected by type 2 DM (Kieler *et al*., 2019). However, data related to DM in cats and dogs are currently limited and therefore further analyses are required to better understand the possible role played by the gut microbiota to cause these metabolic disorders.

## The canine and feline gut microbiota and GI diseases

The lifelong interaction between the complex microbial ecosystem that resides in the GIT and its host plays an essential role in influencing the host health status. Indeed, it has been convincingly demonstrated that a balanced microbial ecosystem is involved in multiple physiological and metabolic functions. Beyond its metabolic role in providing nutritional support to the host by degrading otherwise non‐digestible dietary compounds coupled with the production of various SCFAs and other metabolites that provide nutrients to colonocytes, the gut microbiota is believed to be engaged in a continuous dialogue with the host’s immune system (Honneffer *et al*., 2014). Notably, the intestinal microbial community modulates local and/or systemic immune responses, promotes intestinal barrier integrity, influences bowel homeostasis and functionality, and provides protection against enteropathogen colonization (Pickard *et al*., 2017; Alessandri *et al*., 2019a, 2019b, 2019c; Goto, 2019). The interactions between intestinal bacteria and the host immune system may be regulated either through direct contact between intestinal microorganisms and the innate immune system or indirectly by the release of microbial metabolites. The latter, in turn, can be represented by bacterial products such as SCFAs or vitamins as well as host primary metabolites that can be enzymatically converted into secondary metabolites by (elements of) the intestinal consortia (Suchodolski, 2016). In parallel, in order to ensure intestinal homeostasis, the gastrointestinal mucosa acts as a semipermeable barrier for nutrient absorption and immune sensing on the one hand, while preventing passage of potentially harmful microorganisms, compounds and antigens on the other (Salvo Romero *et al*., 2015). However, when functional or physiological impairments of the intestinal epithelial barrier occur, aberrant consequences such as alteration of intestinal permeability may affect intestinal homeostasis. Similarly, alterations in the abundance or in gut microbiota composition as well as drastic changes in its functional activities may cause dysregulation of the adaptive immune response, and activate an inflammatory process, which in turn may be associated with increased susceptibility to infections. One or a combination of these intestinal perturbations are generally related to the onset of different diseases such as chronic enteropathy (CE), acute haemorrhagic or non‐haemorrhagic diarrhoea syndrome, or even intestinal cancer in dogs and cats (Kieler *et al*., 2017; Gavazza *et al*., 2018; Minamoto *et al*., 2012; Redfern *et al*., 2017). In this context, several markers are considered as clear and common signs of intestinal dysbiosis (Heilmann and Steiner, 2018). Particularly, it seems that an increase of bioavailable oxygen in the intestinal lumen as a consequence of inflammation‐related enhanced intestinal barrier permeability plays a central role in the alteration of the gut microbiota composition in favour of facultative anaerobe proliferation, especially members of the *Enterobacteriaceae* family (Vazquez‐Baeza *et al*., 2016; Zeng *et al*., 2017). Furthermore, intestinal inflammation is commonly associated with bile acid dysmetabolism, i.e. altered bile acid transformation (Duboc *et al*., 2013). Primary bile acids are essential for dietary lipid digestion and absorption, but they also play an important anti‐inflammatory role. Indeed, once primary bile acids reach the hindgut compartments, they may be transformed into secondary bile acids by means of deconjugation and dehydroxylation through metabolic activities elicited by the intestinal microbial community with an accumulation of these secondary metabolites in the intestine where they can exert their anti‐inflammatory properties (Dawson and Karpen, 2015; Suchodolski, 2016). Specifically, while they counteract germination of *C. difficile* spores, they are also able to stimulate induction of GLP‐1, which is involved in increasing the amount of circulating insulin, or to activate the G protein‐coupled receptor TGR5 which, in turn, suppresses the pro‐inflammatory status induced by circulating bacterial cell wall‐associated lipopolysaccharides (LPS) typical of an inflammatory status (Dawson and Karpen, 2015; Blake and Suchodolski, 2016). Therefore, an imbalance in the primary to secondary bile acid ratio is an indication of intestinal dysbiosis with potential negative consequences for host metabolism (Guard *et al*., 2019). In addition, gut dysbiosis is commonly characterized by a significant reduction in the abundance of certain bacterial genera or species involved in the fermentation of complex carbohydrates, resulting in a decreased intestinal concentration of SCFAs, which play an important anti‐inflammatory role and which stimulate differentiation of intestinal helper T (Th) cell precursors into immune regulatory T cells (T_reg_) (Arpaia *et al*., 2013; Suchodolski, 2016). However, despite numerous studies in this field, it is not yet clear whether the functional and microbial taxonomic changes that occur in the intestine are the cause or effect of the inflammatory bowel diseases. This is made even more difficult for dogs and cats since in most cases the diagnosis of inflammatory bowel disease is established after a non‐responsive antibiotic treatment, which in itself will have altered the microbiota composition (Pilla and Suchodolski, 2019). However, investigating the microbial and metabolic alterations will be pivotal to identify potential diagnostic biomarkers in order to facilitate early detection of bowel diseases and to develop therapeutic interventions (Blake and Suchodolski, 2016). In the following sections, the main features of intestinal of both acute and chronic intestinal diseases as well as intestinal cancer of dogs and cats will be briefly covered.

### Acute bowel diseases

The main intestinal diseases for dogs and cats correspond to acute uncomplicated diarrhoea (AD) and acute haemorrhagic diarrhoea syndrome (AHDS; Suchodolski *et al*., 2012a, 2012b,2012a, 2012b). These two intestinal diseases differ in clinical outcomes. Indeed, while AD is characterized by mild symptoms, the clinical consequences of AHDS are more severe with haemorrhagic diarrhoea, dehydration, lethargy and anorexia associated with haemorrhagic lesions in the intestinal mucosa (Unterer *et al*., 2014; Ziese *et al*., 2018). Despite histological and symptomatic differences between the two diseases, AD and AHDS display similar shifts in the intestinal microbial composition (Suchodolski *et al*., 2012a, 2012b,2012a, 2012b; Guard *et al*., 2015). In this context, a study performed on dogs affected by AD or AHDS observed that in both cases the relative abundance of *Blautia*, *Faecalibacterium, Ruminococcus* and *Turicibacter* genera had significantly decreased, while the relative abundance of *Clostridium* spp. was shown to increase when compared to healthy controls (Suchodolski *et al*., 2012a, 2012b,2012a, 2012b). In accordance with these observations, another trial revealed a significant increase in the abundance of *Clostridium* spp., especially *Clostridium perfringens*, in AD or AHDS‐associated canine faecal samples with a simultaneous reduction of the *Prevotella*, *Blautia*, *Faecalibacterium* taxa, *Lachnospiraceae* and *Ruminococcaceae* families (Guard *et al*., 2015). In concert with changes in the taxonomic composition, AD and AHDS are generally associated with shifts in the metabolites produced. In this context, it was observed that acute episodes of diarrhoea not only affect the SCFA profiles, but also serum and urine metabolites, suggesting that acute bowel diseases elicit effects on the overall host‐associated metabolic profile (Guard *et al*., 2015). Specifically, a reduction in propionic acid production was observed in faecal samples of dogs affected by acute diarrhoea, while, although many members of the genera whose abundance decreases in AD or AHDS are known SCFA producers, the total level of butyrate increased. This inconsistency, as the authors suggested, may be the consequence of a reduced absorption or utilization of butyrate by the compromised enterocytes (Guard *et al*., 2015). On the other side, an increased abundance of *Clostridium* spp. was observed in both AD and AHDS diseases (Stoeckel *et al*., 2012; Guard *et al*., 2015; Leipig‐Rudolph *et al*., 2018). Although these species are commensal inhabitants of the GIT of healthy dogs and cats, the pathological role of clostridia in AHDS has recently been demonstrated since clostridia‐like bacteria were found on the necrotic mucosal surface of endoscopic biopsies of dogs with AHDS coupled with an abnormal proliferation of *C. perfringens* (Minamoto *et al*., 2014; Leipig‐Rudolph *et al*., 2018). Further investigations identified the *netF*‐positive type A *C. perfringens* bacterium is responsible for intestinal lesions typical of AHDS. Indeed, the *netF* gene, which encodes a pore‐forming toxin with cytotoxic activities, was shown to be present in the genome of *C. perfringens* strains isolated from different intestinal biopsies of dogs with AHDS (Leipig‐Rudolph *et al*., 2018). Moreover, the observation that recovery from AHDS is generally associated with a significant decrease in the abundance of *netF*‐positive type A *C. perfringens*, further supports the involvement of this toxin in the necrotizing mucosal lesions typical of dogs suffering from this disease (Ziese *et al*., 2018).

Similar to what was observed for dogs, faecal samples of cats with diarrhoea were shown to contain a high abundance of Proteobacteria, especially gamma‐ and beta‐Proteobacteria, *Clostridium* and *E. coli*. However, *C. perfringens* did not show a statistically significant increased relative abundance in cats with AD, thus indicating that this species does not play a significant role in feline GI diseases (Suchodolski *et al*., 2015). Instead, a role of *E. coli* in episodes of feline diarrhoea was proposed, given its statistically significant increase and its involvement in other GI diseases (Suchodolski *et al*., 2015). However, further studies are required to evaluate the precise role of this bacterial species as a microbial marker of dysbiosis or as an enteropathogen that contributes to intestinal diseases in cats. It should be noted that certain other taxa, such as *Faecalibacterium* and *Roseburia*, were shown to elicit decreased relative abundance in faecal samples of cats suffering from AD (Suchodolski *et al*., 2015).

### Chronic intestinal diseases

The term ‘chronic enteropathy’ (CE) refers to a heterogeneous group of intestinal disorders that are generally classified in accordance to their response to treatment, encompassing diet‐responsive enteropathy (FRE), antibiotic‐responsive enteropathy (ARE) and immunosuppressant‐responsive enteropathy (IRE) better known as idiopathic inflammatory bowel diseases (IBD; Dandrieux, 2016). Despite having different aetiologies, CE disorders in dogs and cats are characterized by the overlap of persistent and recurrent clinical signs, including histological evidence of intestinal inflammation as well as vomiting, diarrhoea, hyporexia, abdominal pain and weight loss (Dandrieux, 2016). Furthermore, CEs occur spontaneously with similar multifactorial aetiology considering a combination of genetic susceptibility of the host, aberrant host immune system, dietary and/or environmental factors and altered intestinal microbial ecosystem (Minamoto *et al*., 2012; Vazquez‐Baeza *et al*., 2016). Although the involvement of the intestinal microbiota in the aetiology of CE is widely accepted, it is still difficult to define specific microbial biomarkers that can be directly associated with these intestinal disorders, due to the lack of a standard protocol for the study of the intestinal microbial community (Redfern *et al*., 2017). However, some common features have been identified. In dogs with CE, the relative abundance of Fusobacteria as well as some representatives of the Bacteroidetes phylum, i.e. Bacteroidaceae and Prevotellaceae decrease compared with healthy controls (Suchodolski *et al*., 2012a, 2012b,2012a, 2012b). Similarly, the relative abundance of several members of the Firmicutes phylum declines in dogs with CE, including *Megamonas*, *Ruminococcus*, *Faecalibacterium*, *Blautia* and *Turicibacter* and *Lachnospiraceae* family (Suchodolski *et al*., 2012a, 2012b,2012a, 2012b; Minamoto *et al*., 2015, 2019; Xu *et al*., 2016). Conversely, certain members of the Proteobacteria phylum and specifically the *Enterobacteriaceae* family, including in particular *E. coli*, were shown to elicit a significant relative abundance increase in dogs with CE (Suchodolski *et al*., 2012a, 2012b,2012a, 2012b; Minamoto *et al*., 2015; Xu *et al*., 2016).

In accordance to what has been observed in dogs, a study involving 16S rRNA microbial profiling revealed that bacterial taxa belonging to the *Ruminococcaceae* family and to the *Turicibacter* genus are significantly less abundant in cats with CE than in healthy controls (Marsilio *et al*., 2019). In the same way, some members of the Bacteroidetes, especially *Bacteroides plebeius* which has been associated with remission in humans with inflammatory bowel disease, and Actinobacteria phyla showed a trend towards a decrease abundance in case of CE (Marsilio *et al*., 2019). In contrast, representatives of the *Enterobacteriaceae* and *Streptococcaceae* families appear to increase in CE‐associated feline faeces coupled with *Desulfovibrio* spp., which are known to be toxic sulfide producers (Inness *et al*., 2007; Marsilio *et al*., 2019). Furthermore, various studies, employing different experimental procedures, including 16S rRNA sequencing and fluorescence *in situ* hybridization (FISH), highlighted that cats with CE generally show a decrease in the relative abundance of the *Bifidobacterium* genus, a trend that had not been documented for dogs suffering from CE (Inness *et al*., 2007; Marsilio *et al*., 2019).

As expected from changes in the taxonomic composition, a dysbiotic gut microbiota is also characterized by functional shifts with consequent impacts on the production of bacterial metabolites. Several SCFA‐producing bacteria such as *Ruminococcus*, *Faecalibacterium*, *Turicibacter* and *Lachnospiraceae* family significantly decreased in dogs and cats with CE. In this context, it has been demonstrated that faecal concentration of total SCFAs was significantly lower in dogs with CE than in the control group. Further scrutiny of the SCFA profile revealed that while butyrate showed only a decreasing trend, acetate and propionate levels were significantly reduced, implying an impact on the host immune system since SCFAs possess anti‐inflammatory features (Minamoto *et al*., 2019). Particularly, propionate is known to inhibit pro‐inflammatory cytokine production such as IL‐6, IL‐8 and TNF‐α (Moylan *et al*., 2020) and to simultaneously stimulate the expression of the anti‐inflammatory cytokine IL‐10 coupled with the Foxp3 transcriptional factor which is crucial for regulation of intestinal inflammation (Smith *et al*., 2013). In this context, a reduction of the number of Foxp3‐positive T_reg_ cells was recorded in the duodenal mucosa of dogs with IBD, suggesting that a decrease in intestinal propionate levels plays a role in the pathogenesis of IBD in dogs (Maeda *et al*., 2016). In addition to alterations in faecal SCFA levels, CE in dogs is associated with decreased amino acid metabolism, indicating that CE‐associated gut microbiota is responsible for dysfunctional protein metabolism in the presence of intestinal inflammation (Minamoto *et al*., 2015). Furthermore, alteration in serum metabolite profile was pointed out as a typical sign of CE in both dogs and cats (Minamoto *et al*., 2015; Xu *et al*., 2016; Sakai *et al*., 2018). Notably, significantly lower levels of circulating tryptophan were detected in dogs and cats with CE, encompassing dogs with protein‐losing enteropathy, which is a particular form of CE characterized by hypoproteinaemia due to a drastic loss of protein in the intestinal tract (Kathrani *et al*., 2018; Sakai *et al*., 2018; Tamura *et al*., 2019). Tryptophan is an essential amino acid involved in protein synthesis and acts as a precursor for several bioactive compounds such as serotonin, melatonin and kynurenine (Richard *et al*., 2009). In addition, tryptophan may be used by intestinal bacteria in order to produce a range of indole compounds involved in activating anti‐inflammatory pathways (Lavelle and Sokol, 2020). Altogether, these findings highlight that tryptophan plays an important role in intestinal inflammatory diseases.

### Bowel cancer

Besides acute or chronic gut diseases, alterations of the intestinal homeostasis are also believed to be involved in colonic carcinogenesis (Grzeskowiak *et al*., 2015; Gavazza *et al*., 2018). In fact, several studies have incriminated bacterial‐induced chronic bowel inflammation as promoter of a tumour‐permissive environment characterized by intestinal bacteria translocation into the circulatory system as well as mucosal infiltrations of tumour progression‐related cells (Garraway *et al*., 2018). Furthermore, in humans, a dysbiotic microbiota triggers a series of innate and adaptive immune responses involved in tumour genesis as well as the production of microbial metabolites such as lipoteichoic acids that through their binding to Toll‐like receptor 2 causes excessive secretion of pro‐inflammatory compounds or secondary bile acids that in this case can be detrimental by activating G protein‐coupled bile receptor 1, by promoting intestinal cell proliferation, DNA damage, cellular senescence and ultimately carcinogenesis (Meng *et al*., 2018). Although the relationship between intestinal dysbiosis and colorectal cancer has been widely debated in humans, only a small number of studies have been focused on the correlation between bowel cancer and gut microbiota in dogs and cats and, what is more, with discordant results (Omori *et al*., 2017; Garraway *et al*., 2018; Gavazza *et al*., 2018; Herstad *et al*., 2018). However, while the relative abundance of the *Fusobacterium* genus and the *Enterobacteriaceae* family increased in ileum and colon biopsies from cats with small cell GI lymphoma (Garraway *et al*., 2018), no differences of the abundance of these two bacterial taxa were observed in dogs affected by intestinal lymphoma when compared to the control (Omori *et al*., 2017; Gavazza *et al*., 2018; Herstad *et al*., 2018) and even one study reported a decreased abundance of *Fusobacterium* spp. in canine lymphoma biopsies (Gavazza *et al*., 2018). Conversely, while *Faecalibacterium* seems to decrease in case of canine lymphoma, the *Streptococcus* genus showed an opposite trend (Gavazza *et al*., 2018; Herstad *et al*., 2018). Despite these observations, further studies are required to comprehensively understand the implications of the gut microbiota in the onset of intestinal tumours in dogs and cats.

## Therapeutic strategies for the treatment of inflammatory bowel diseases

In recent years, several treatments have been tested to try to restore the intestinal homeostasis in case of CE by manipulating the intestinal bacterial community. Antibiotics, prebiotics, probiotics, synbiotics, corticosteroids or even, though in exceptional cases, faecal microbiota transplantation (FMT) have been employed as therapeutic treatments (Manchester *et al*., 2019; Pilla *et al*., 2019; Sugita *et al*., 2019). However, there is no standard treatment that offers the best strategy to adopt for this scope. Indeed, typical treatments involve sequential trials starting with the less invasive diet, followed by antibiotics and ultimately immune‐suppressive drugs in non‐responsive dogs or cats (Dandrieux, 2016; Dandrieux *et al*., 2019).

Antibiotics have long been considered to represent the first port of call and indeed the gold standard for treatment of acute or chronic intestinal inflammation, and antibiotics are still considered one of the main components of sequential therapy for dogs and cats with CE. However, if the effectiveness of the use of antibiotics has been reported in case of infections, their real benefit to treat CE is uncertain since antibiotics are surrounded by many contradictions. Indeed, the employment of antibiotics can expose animals to risk factors such as a significant decline of the intestinal microbial biodiversity, reduction of beneficial bacteria and development of antibiotic‐resistant microorganisms (Suchodolski *et al*., 2009; Dandrieux *et al*., 2019; Werner *et al*., 2020). Metronidazole and/or tylosin are the most commonly prescribed antibiotics to treat GI diseases (Mondo *et al*., 2019). However, although several trials highlighted remission of dogs and cats after metronidazole or tylosin treatments, these antimicrobials are often administered in combination with dietary therapy or other drugs, thus preventing a complete understanding of the true impact of antibiotics in CE treatment (Munster *et al*., 2006; Makielski *et al*., 2019). In this context, the administration of prednisone (a corticosteroid) to dogs with IBD resulted as effective as the combined treatment with both prednisone and metronidazole (Jergens *et al*., 2010). In a similar way, the treatment with amoxicillin/clavulanic acid did not reduce mortality rate, duration of hospitalization or severity of clinical signs in dogs with AHDS, thus emphasizing the marginal ole of antibiotics in counteracting CE (Unterer *et al*., 2011). Furthermore, tylosin treatment induced a significant decrease in commensal taxa such as *Fusobacterium*, *Faecalibacterium*, *Blautia* and *C. hiranonis*, while *Enterococcaceae* and *Peptostreptococcaceae* increased probably due to their intrinsic or acquired antimicrobial resistance, thus arguing against the hypothesis that tylosin may elicit a beneficial effect in the restoration of a dysbiotic microbiota (Manchester *et al*., 2019). In addition, different studies reported that CE response to antibiotics is short‐lasting after cessation of the treatment with a high number of relapsing cases within a month (Dandrieux *et al*., 2019). Based on these findings, other solutions such as prebiotics, probiotics or synbiotics may be preferred for CE treatment.

As already mentioned in this review, prebiotics are non‐digestible compounds (frequently represented by fibres or carbohydrates) that promote proliferation of beneficial bacteria residing in the GIT of the host (Gibson *et al*., 2004). Probiotics, instead, are live microorganisms that, when consumed in adequate amounts, are able to confer health benefits to the host (Grzeskowiak *et al*., 2015). Among the various mechanisms of action, probiotics may exert their health benefits by inhibiting pathogenic bacteria by competition for nutrients or mucosal adhesion sites, by improving the intestinal barrier functions or by enhancing the immune responses (Sanchez *et al*., 2017). Synbiotics are formulated as a combination of synergistically acting probiotics and prebiotics aimed at promoting the survival and implantation of exogenous live microorganisms (i.e. the probiotic) through the supply of specific carbohydrates or fibres (representing the prebiotic; Gibson and Roberfroid, 1995; Markowiak and Slizewska, 2017). However, although several studies reported the beneficial effects of probiotics‐ and/or synbiotics‐based therapies to canine with CE (Table 2), no increase in the relative abundance of the administered microorganisms occur, thus suggesting that probiotic or synbiotic treatments have only negligible and transient effects on faecal microbial community (Garcia‐Mazcorro *et al*., 2011; Larsen *et al*., 2011; Rossi *et al*., 2014). For this reason, probiotic administration may be associated with standard immunosuppressive treatment.

**Table 2:** Effects of probiotic or symbiotic administration to dogs with CE.

Treatment	Type of disorder	Effect of the treatment	References
High‐fibre diet with probiotic blend	FRE	Resolution of clinical signs with improvement of faecal scores and Canine Chronic Enteropathy Clinical Activity Index (CCECAI) and histologic amelioration.	Rossi *et al*. (2020)
*Enterococcus faecium* NCIMB 10415 E1707 with FOS and gum Arabic	FRE	No differences in clinical efficacy, histologic scores or expression of specific cytokines emerged after the treatment.	Schmitz *et al*. (2015)
*Enterococcus faecium* NCIMB 10415 E1707 with FOS and gum Arabic	FRE	Increased intestinal biodiversity coupled with a slight increment of the *Enterococcus* genus relative abundance	Pilla *et al*. (2019)
Sour milk with three canine‐derived *Lactobacillus* strains	AD	Normalizing effects in stool consistency and improvement of the animal conditions with reduced vomiting and increased appetite. Remarkable reduction of *C. perfringens* alphatoxin‐ or enterotoxin‐producing strains	Gomez‐Gallego *et al*. (2016)
Probiotic VSL#3	IBD	Increased relative abundance of the *Faecalibacterium* genus and improvement of histological scores with enhancement of regulatory T‐cell markers (FoxP3+ and TGF‐β)	Rossi *et al*. (2014)
Multi‐strain probiotic	IBD	Increased relative abundance of *Lactobacillus* spp., rapid clinical remission and increased expression of tight junction proteins	White *et al*. (2017)

Although several studies have been carried out to understand the impact of probiotic/synbiotic‐based therapies on dogs with CE, currently available literature regarding such studies in cats is extremely scarce. Indeed, only a single study reported the use of a probiotic to treat intestinal disorders such as chronic constipation or idiopathic megacolon (Rossi *et al*., 2018). In this case, the administration of a probiotic blend resulted in significant clinical improvements coupled with reduced mucosal infiltration and enhanced histological parameters suggesting an anti‐inflammatory effect of the probiotic blend (Rossi *et al*., 2018). In light of this data and considering the benefits observed in dogs with CE, it is plausible to suggest that also in cats, treatments with probiotics or synbiotics may result in beneficial effects in case of intestinal bowel diseases. However, large‐scale trials are needed in order to support this hypothesis.

A newly emerging experimental frontier in the treatment of human intestinal diseases is represented by FMT, a procedure that aims to restore the dysbiotic gut microbiota of a patient affected by intestinal diseases by endoscopically or colonoscopically administering the faecal matter from a healthy donor (Chaitman *et al*., 2016). In human medicine, FMT has been successfully employed to treat recurrent and refractory *C. difficile* infections. In this situation, FMT is currently considered one of the most effective and safe solutions to eradicate *C. difficile* infection when compared to antibiotic therapies (Seekatz *et al*., 2014; Quraishi *et al*., 2017; Hui *et al*., 2019). Furthermore, the application of this procedure in other clinical settings, such as human IBD, has also resulted in beneficial outcomes for patients (Blanchaert *et al*., 2019). Based on these findings, FMT trials have been successfully performed in the veterinary field for the treatment of intestinal diseases in dogs and cats (Pereira *et al*., 2018; Niina *et al*., 2019; Sugita *et al*., 2019), resulting in successful recovery of a cat with IBD (Furmanski and Mor, 2017), faster resolution of diarrhoea and shorter hospitalization of puppies affected by parvovirus infection (Pereira *et al*., 2018), complete remission of both a dog affected by intermittent large bowel diarrhoea and *C. difficile* antigen and toxin A&B genes and proteins in its faecal sample and a dog with a prolonged history of vomiting and diarrhoea (Niina *et al*., 2019; Sugita *et al*., 2019).

Despite such promising outcomes, further clinical trials are required to develop a reliable protocol for a standardized FMT treatment in dogs and cats. Indeed, several factors that may influence the success of the FMT treatment should be taken into consideration, including the preservation of the donor faecal sample, timing of administration (single or multiple treatment) and the method of faecal supplementation (endoscopy, nasogastric tube, capsules, colonoscopy or retention enema).

## The impact of human–pet interplay on gut microbiota composition of both humans and companion animals

Humans have been identified as major players in driving several evolutionary events, encompassing extinction or speciation through domestication, relocation and creation of novel ecosystems (Bull and Maron, 2016). Specifically, through domestication, humans have deliberately altered feeding, behaviour, habitat and genetic heritage of multiple animal species (Wang *et al*., 2013; Frantz *et al*., 2020). During the course of evolution, dogs and cats were among the first animals to have undergone a long and extensive domestication process starting from their direct ancestors, the grey wolf and wild cat, respectively, leading to the selection of a wide range of canine and feline breeds (Savolainen *et al*., 2002; Baca *et al*., 2018). However, apart from animal phenotypic and genotypic alterations, several studies have demonstrated that the anthropogenic influence has significantly modified the intestinal microbiota of domesticated animals when compared to their close, yet wild relatives (Metcalf *et al*., 2017; Alessandri *et al*., 2019a, 2019b, 2019c). In this milieu, dietary shifts, antibiotic exposure, reduced contact with nature and a concomitant close interaction with humans are only some of the multiple substantial changes imposed by domestication that may have impacted on the intestinal microbial community of animals (Ferrario *et al*., 2017; McKenzie *et al*., 2017). A recent study investigated the effects of artificial selection and close contact with humans on canine gut microbiota, comparing the 16S rRNA gene sequencing‐based core gut microbiota of dogs with that of humans and wolves (Alessandri *et al*., 2019a, 2019b, 2019c). This comparison highlighted that while six bacterial genera belonging to the core gut microbiota of wolves, i.e. *Alistipes*, *Pseudomonas*, *Slackia*, *Subdoligranulum*, *Eubacterium coprostanoligenes* and *Barnesiella,* have apparently been lost by the domesticated canine core gut microbiota, the latter have acquired five other microbial taxa, including *Dorea*, *Parabacteroides*, *Streptococcus*, U. m. of Bacteroidales order and U. m. of Clostridiales order, which are typical components of the human core intestinal microbiota (Alessandri *et al*., 2019a, 2019b, 2019c). These findings suggest that the shift from a natural and undomesticated lifestyle to cohabitation with humans has modulated the intestinal microbial community of domesticated dogs through horizontal transmission events. To support this observation, comparison of the gut microbiota of wolves with that of domestic dogs revealed that while the *Streptococcus* genus was absent in the intestinal community of wolves, it was detected in the canine one (Wu *et al*., 2017). Furthermore, representatives of the Cyanobacteria phylum were exclusively present in wolves, while members of the Verrucomicrobia taxa were found only in canine faeces (Wu *et al*., 2017). This strengthens the notion that human interference has played a role in the modulation of canine gut micro‐ecology. Moreover, it has been demonstrated that changes in feeding habits due to domestication has led to the differentiation in both composition and functional activities of the gut microbiota of dogs when compared to wolves (Lyu *et al*., 2018). Specifically, some bacterial taxa associated with a complex polysaccharide‐rich diet, including *Ruminococcaceae*, *Desulfuromusa*, *Lactobacillus*, *Carnobacillus* and *Faecalibacterium,* showed a significantly higher relative abundance in dogs, thus indicating that the composition of the canine gut microbiota has been influenced by dietary changes imposed by humans (Lyu *et al*., 2018). To further support this notion, it was observed that the canine gut microbiome is enriched in genes involved in carbohydrate metabolism pathways as well as genes encoding for GHs when compared to that present in wolves (Lyu *et al*., 2018).

However, cohabitation and the resulting close relationship between humans and their pets have not only significantly influenced the animal microbiota, but also the human‐associated microbial community (Song *et al*., 2013). In particular, it has been demonstrated that dog ownership influences the human skin microbiota. Indeed, the presence of a dog within a family generally leads to a higher microbial biodiversity of the adult skin, including hands and forehead, when compared to adults without dogs (Song *et al*., 2013). Furthermore, it seems that dogs may act as carriers of bacteria since not only dogs and their owners share various bacterial phylotypes between fur and skin, but it was also highlighted that adults that live together and simultaneously possess a pet share more skin bacteria than a couple without pets (Song *et al*., 2013). Pet ownership does not only impact on the adult microbial communities, but it also appears to play a role in modifying the infant gut microbiota. Indeed, a recent study showed that pre‐ and/or post‐natal exposure to household pets influences the infant gut microbiota (Tun *et al*., 2017). In general, regardless of birth variables, including type of delivery or intrapartum antibiotic prophylaxis, pet exposure induces a significant increase in species richness in the Firmicutes phylum, especially the *Ruminococcus* and *Oscillospira* genera, in the infant intestinal microbial community (Tun *et al*., 2017). In detail, while *Oscillospira* is associated with leanness and lower body mass index in both adults and infants, *Ruminococcus* is linked to the maintenance of the intestinal barrier integrity (Tun *et al*., 2017). Furthermore, both *Ruminococcus* and *Oscillospira* have been negatively related to the development of infant atopy and obesity, respectively, suggesting that pet‐associated microbiota may have a role in modulating the infant gut microbiota (Tun *et al*., 2017). In addition, it has been reported that perinatal pet exposure may impact on the composition of the infant intestinal microbiota and solicit the immune responses, thus inducing protection against infant wheezy bronchitis (Nermes *et al*., 2013). Despite the abovementioned positive influence of vertical transmission in dogs and cats, these animals may also act as a reservoir for opportunistic pathogens, such as *Escherichia coli*, *Salmonella* and *Campylobacter* that may be transmitted horizontally, and may thus cause zoonotic enteric diseases to humans (Morato *et al*., 2009; Lambertini *et al*., 2016; Moon *et al*., 2018). At the same time, the pet intestinal ecosystem may be a vector of antimicrobial resistance, thus representing a serious global health safety issue (De Graef *et al*., 2004). Overall, these results show that cohabitation and the close relationship between pet and their owners play an important, yet varied role in gut microbiota modulation with repercussions on the health of both parties.

## Conclusions

During the course of evolution, dogs and cats have become the main companion animals for humans. Being an integral part of human life, interest for pet health and well‐being has rapidly increased during recent decades and has consequently promoted many studies concerning the canine and feline gut microbiota, which are known to influence the health status of the host. In this perspective, it has been demonstrated that diet, age, anthropogenic influences and several other environmental factors play a role in modulating both the taxonomical composition and the functional activities of the intestinal microbial community of dogs and cats. Furthermore, alterations of the intestinal homeostasis are generally associated with a dysbiotic gut microbiota and the onset of inflammatory bowel diseases that may be treated with different therapies, including prebiotics, probiotics, synbiotics, antibiotics or FMT as for humans IBD. Nevertheless, further research is necessary to deepen our understanding of the role of this microbial community and to better appreciate how it interacts with dogs and cats.

## Conflict of interest

The authors declare that they have no competing interests.
